# Ethnodemographic characterization of stroke incidence and burden of disease in hospital discharge records in Ecuador

**DOI:** 10.3389/fneur.2023.1059169

**Published:** 2023-02-08

**Authors:** Sarah J. Carrington, Daniel Romero-Alvarez, Marco Coral-Almeida, Andrea Vela, Aquiles Rodrigo Henríquez-Trujillo, Guido Mascialino

**Affiliations:** ^1^Department of Economics, Universidad de Las Américas, Quito, Ecuador; ^2^Biodiversity Institute and Department of Ecology and Evolutionary Biology, The University of Kansas, Lawrence, KS, United States; ^3^One Health Research Group, Faculty of Medicine, Universidad de Las Américas, Quito, Ecuador; ^4^Grupo de bioquimioinformática GBQ, Universidad de Las Américas, Quito, Ecuador; ^5^Facultad de Ciencias Pecuarias, Carrera de Medicina Veterinaria, Escuela Superior Politécnica de Chimborazo, Chimborazo, Ecuador; ^6^Coordinación General de Sostenibilidad del Sistema y Recursos, Ministerio de Salud Pública, Quito, Ecuador; ^7^Escuela de Psicología, Universidad de Las Américas, Quito, Ecuador

**Keywords:** stroke, ethnicity, gender, disability-adjusted life years (DALYs), Ecuador

## Abstract

**Introduction:**

Stroke is the second most common cause of death and disability-adjusted life years (DALYs) globally. However, the incidence and impact of stroke by ethnicity and gender is frequently distinct. This is particularly the case in Ecuador where geographic and economic marginalization are often correlated with ethnic marginalization and the extent to which females lack the same opportunities as their male counterparts. The aim of this paper is to investigate the differential impacts in terms of stroke diagnosis and burden of disease by ethnicity and gender, using hospital discharge records over the years 2015–2020.

**Methods:**

This paper calculates stroke incidence, and fatality rates using hospital discharge and death records over the years 2015–2020. The DALY package in R was employed to calculate the Disability Adjusted Life Years lost due to stroke in Ecuador.

**Results:**

The results show that while the incidence rate of stroke in males (64.96 per 100,000 persons-year) is higher than that for females on average (57.84 per 100,000 persons-year), males accounted for 52.41% of all stroke cases and 53% of all surviving cases. Thus, hospital data suggests that females had a higher death rate when compared to males. Case fatality rates also differed significantly by ethnicity. The highest fatality rate corresponded to the Montubio ethnic group (87.65%), followed by Afrodescendants (67.21%). The estimated burden of disease of stroke calculated using Ecuadorian hospital records (2015–2020) varied from 1,468 to 2,991 DALY per 1,000 population on average.

**Discussion:**

Differences in the burden of disease by ethnic group are likely to reflect differential access to care by region and socio-economic group, both of which are frequently correlated with ethnic composition in Ecuador. Equitable access to health services remains an important challenge in the country. The gender discrepancy in fatality rates suggests that there is a need for targeted educational campaigns to identify stroke signs early, especially in the female population.

## Introduction

Stroke is the second most common cause of death and disability-adjusted life years (DALYs) globally (1, 2). The worldwide prevalence of stroke almost doubled from 1993 to 2010, and its burden of disease also increased during that time period (3). Although stroke has been rightly considered a global health crisis (4), its impact has been greater in developing countries (3). Johnston et al. (5) found that national per capita income was the best predictor of mortality and DALYs, noting a ten-fold difference between lowest and highest rated nations. Moreover, over the last two decades there has been an increase in burden caused by stroke in developing countries (from 0.9–2.1 DALYs in 1991 to 1.7–2.8 DALYs in 2013) (3). In Latin America, the growing prevalence of stroke has led Del Brutto et al. (6) to note that stroke is becoming a public health problem.

In addition to the well-documented disparity in the geographical impact of stroke, racial/ethnic disparities in the epidemiology, treatment, and health outcome of stroke have been demonstrated (7–9). Research on ethnic/racial disparities has taken place almost exclusively in the United States, where African-Americans have been found to have a higher incidence of strokes, higher mortality rates, and poorer long-term outcomes when compared to Whites (9–12). Research examining ethnic/racial disparities in other countries is scarce. Evidence of higher rates of small vessel disease in black patients compared to white patients was found by Markus et al. (13) in London. In a stroke registry in Argentina, a higher frequency of hemorrhagic strokes for South American Natives compared to Whites was noted (14). Del Brutto et al. (6) determined the prevalence of intracranial arterial stenosis in Ecuadorian Natives/Mestizos and found it comparable to that of other minorities as obtained from various studies in other countries.

In addition to racial/ethnic disparities in stroke outcomes, studies have documented a gender effect. A systematic review of mortality and gender in stroke found higher in-hospital crude mortality for women, which disappeared in adjusted models (15). A similar review of stroke epidemiology (16) revealed a higher incidence and prevalence for men, while stroke severity was higher in women, reflecting a higher case fatality rate (24.7 vs. 19.7%). Furthermore, in certain conditions, such as atrial fibrillation, women are at an increased risk of stroke (17). Symptom presentation may vary by gender (18) as well as prehospital delay in some regions (19) with women having a longer delay before hospitalization.

Research devoted to stroke in Ecuador is scant but has increased significantly in the last 5 years. A recent retrospective analysis of a health registry with 77,897 cases, spanning 1991-2015, revealed stroke has consistently been the number one cause of mortality in Ecuador throughout those years (20). The Atahualpa Project, (6) a community-based prospective study of stroke incidence in rural areas, found that stroke prevalence for people aged 40 and above in that area grew from 4.08% in 2003 to 31.15% in 2012.

Ecuador is an ethnically and racially diverse country with sizeable Indigenous, Mestizo, Afro-descendant, Montubio and White populations (21). In spite of this diversity, as well as the growing prevalence of stroke in Ecuador, there is no research exploring the ethnic/racial disparities of stroke in this country. The current study aims to fill that vacuum by comparing the prevalence of stroke diagnosis in hospital discharge records between different ethnic/racial groups. Furthermore, given documented epidemiological differences in stroke by gender, differences in this group will also be explored. Finally, life expectancy and burden of diseases in ethnic/racial groups will be determined.

## Methods

A retrospective analysis was conducted of a publicly available database created by the Instituto Nacional de Estadística y Censos (22), a governmental entity responsible for tracking various health metrics at the national level. Anonymized data regarding hospital discharge diagnosis, as well as associated demographic data, is collected for all health institutions, both public and private. Age, gender, ethnic/race (self-identified), and discharge diagnoses were abstracted from the database for the years 2015–2020. Discharge diagnoses were coded in the database using the International Classification of Diseases 10th Revision (ICD-10). Diagnoses classified as stroke were: I60: Subarachnoid hemorrhage, I61: Intracerebral hemorrhage, I62: Other nontraumatic intracranial hemorrhage, I63: Cerebral infarction, and I64: Stroke, not specified as hemorrhage or infarction. Patients self-identified as either Indigenous, Afro-descendant, Mestizo, Muntubio, or White. Patients who identified as other or those who did not provide ethnic/racial identification were excluded from the analysis. Rates of stroke diagnosis by ethnicity were obtained by dividing stroke diagnosis for each ethnic group by total discharges.

## Study population

The Ecuadorian population includes an estimated 17,510,643 inhabitants as of 2020, with the last nationwide census recording 15,012,228 inhabitants in 2010 (23). Of these inhabitants, the Instituto Nacional de Estadística y Censos (henceforth INEC) distinguishes between six self-identified ethnic groupings, these being: “Indigenous”, “Afrodescendent”, “Montubio”, “Mestizo”, “White” and a residual group denoted “Other” (24). As of 2020, it is estimated that the ethnic composition of Ecuador is as shown in Table 1, and that this reflects the steady proportional composition of the population over time.

**Table 1:** Population proportion by self-declared ethnicity in Ecuador according to the estimations of the Instituto Nacional de Estadística y Censos (INEC) for 2020.

**Ethnicity**	**Population proportion**
Indigenous	7.03%
Afrodescendent	7.19%
Montubio	7.39%
Mestizo	71.93%
White	6.09%
Other	0.37%

### Sources of information

This study uses annual data over the period 2015–2020 taken from the Registries of all hospital deaths and hospital admissions resulting in discharges or decease reported at the national level by the General Direction of Civil Registry and the Ecuadorian Ministry of Health. These registries are collected by INEC (22, 25) and the data used in this study was downloaded from these consolidated databases. The international ICD codes I60-64 were used to identify deaths and hospital admissions due to Strokes. Data were processed in Microsoft Excel before using the DALY calculator in R v4.1.2.

### Estimation of the burden of disease

The burden of disease attributable to stroke during the study period was measured using Disability Adjusted Life Years (DALY): the sum of years lived with disability (YLDs) and years of life lost due to premature mortality (YLLs), following the methods described by Murray et al. (26–29) for the Global Burden of Disease (GBD) studies. Calculations were made using the “DALY” package for R (30).

YLLs were estimated as the product of the number of deaths registered due to stroke in the study period, and the residual life expectancy at the age of death. Residual life expectancy was estimated as per the R program (it uses GBD 2010 and has a value of 86.02 years for females and males). For DALY calculations we used 3 different specifications: the first without a time discount rate and without age weighting, the second with a 3% per year time discount rate to reflect the preference for life years closer to the present, but without age weighting; and the third with both the 3% per year time discount rate as well as age weighting (26, 31).

Available data from hospital admissions registered by the public healthcare services are only a subset of the symptomatic stroke population with effective access to healthcare and not suitable for YLD estimations at the national level.

Disability weights (DW) from the estimates from the GBD 2019 study^1^ for all stroke types range from 0.019 for mild stroke to 0.588 for severe stroke with cognitive problems as a result. As the severity of stroke of the patients within the hospital record sample is not known, an average of the disability weights of the five levels of stroke severity is taken and applied to all cases, which comes to 0.24825.^2^ While using the average disability weight for stroke is not necessarily representative of the actual severity of the condition of the stroke patients, as we do not have information otherwise, this is arguably the least biased assumption. This DW was used for the YLD calculations. The age-segregated data infers that the incidence rate of hospital stroke cases ranges from, on average, 19 per 100,000 person years for infants of both sexes and 2,353 (males) and 2,176 (females) per 100,000 people for adults 60 years of age and over within the population. These measures vary by ethnicity, however. Ethnic differences may be due to innate risk factors that differ by genetics as well as differential access to health and diagnostic services that is often correlated with ethnicity in Ecuador where income, geographic distribution, education, and essential service access are often correlated with ethnic attributes (34–36). Table 2 describes the parameters used for burden estimations and their probability distributions used in the DALY package for R.

**Table 2:** Parameters and probability distributions used for calculation of disability-adjusted life years (DALYs).

**Parameter**	**Probability distribution**	**Value range**	**Source**
Population	Fixed by age and sex	15.0 in 2010–17.5 million in 2020 inhabitants	INEC (23)
Prevalence of stroke	Fixed	19–2,353 per 100,000 people	Calculated from data consolidated by INEC (22, 24)
Disability Weight average of severity levels 1–5	Fixed	Average DW = 0.24825 (Range from 0.019 to 0.588)	Calculated from data taken from Institute for Health Metrics and Evaluation (IHME) (32) Global Burden of Disease Study 2019 (33)
Assumed average duration of disability in years (males and females).	Fixed	1 year	
Treatment proportion of male and female	Fixed	1 [range 0–1].	Based on that the data used all being treated cases.
Male incidence–age group 0–4 years	Fixed	20 per 100,000 males in total population (0–27 per 100,000 males in total population when separated by ethnicities).	Calculated from data consolidated by INEC (22, 24)
Male incidence–age group 5–14 years	Fixed	18 per 100,000 males in total population (1–25 per 100,000 males in total population when separated by ethnicities).	Calculated from data consolidated by INEC (22, 24)
Male incidence–age group 15–44 years	Fixed	107 per 100,000 males in total population (2–144 per 100,000 males in total population when separated by ethnicities).	Calculated from data consolidated by INEC (22, 24)
Male incidence–age group 45–59 years	Fixed	490 per 100,000 males in total population (19–655 per 100,000 males in total population when separated by ethnicities).	Calculated from data consolidated by INEC (22, 24)
Male incidence–age group 60+ years	Fixed	2,353 per 100,000 males in total population (70–3,140 per 100,000 males in total population when separated by ethnicities).	Calculated from data consolidated by INEC (22, 24)
Female incidence–age group 0–4 years	Fixed	19 per 100,000 females in total population (0–24 per 100,000 females in total population when separated by ethnicities).	Calculated from data consolidated by INEC (22, 24)
Female incidence–age group 5–14 years	Fixed	15 per 100,000 females in total population (0–19 per 100,000 females in total population when separated by ethnicities).	Calculated from data consolidated by INEC (22, 24)
Female incidence–age group 15–44 years	Fixed	78 per 100,000 females in total population (5–102 per 100,000 females in total population when separated by ethnicities).	Calculated from data consolidated by INEC (22, 24)
Female incidence–age group 45–59 years	Fixed	420 per 100,000 females in total population (17–554 per 100,000 females in total population when separated by ethnicities).	Calculated from data consolidated by INEC (22, 24)
Female incidence–age group 60+ years	Fixed	2,176 per 100,000 females in total population (62–2,911 per 100,000 females in total population when separated by ethnicities).	Calculated from data consolidated by INEC (22, 24)

### Statistical analysis

Descriptive statistics for all variables were obtained. Pearson's Chi-squared, Chi-squared for given proportions, Chi-squared with Yate's correction and parametric/non-parametric ANOVAs were utilized, when appropriate, to compare differences in age, gender, and ethnicity/race outcomes in hospital stroke diagnoses. Bonferroni and Tukey *post-hoc* tests were applied when necessary. Statistical significance was considered with *p*-values < 0.05. 95% Poisson confidence intervals were applied to report incidences. All analysis were conducted in R version 4.1.3 and Excel.

## Results

During the period of 2015–2020, 62,218 cases of stroke were recorded in Ecuador with 18,712 cases resulting in death and 43,506 hospital discharges. The mean age for hospital discharges was of 65.31 with a median of 69 years old, and a standard deviation (st.dev) of 18.89. The mean age for deaths from stroke was 71.5 years with a median of 75 and a st.dev of 19.5 years.

**Figure 1:**
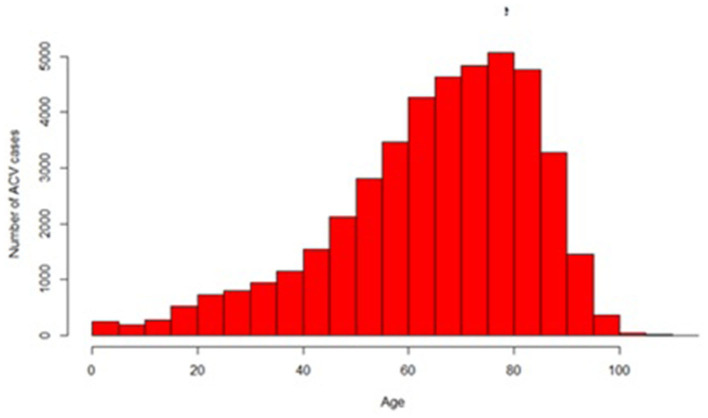
Distribution of stroke cases by age in Ecuador between 2015 and 2020.

Stroke Cases distributed by age are displayed in Figure 1.

### Gender characterization of stroke

For the 2015–2020 period, the incidence rate in males was 64.96 per 100,000 persons-year [64.26–65.67] 95% CI, and the incidence rate in females was 57.84 per 100,000 persons-year [57.18–58.50] 95% CI. The incidence rate in males was significantly higher than in women (*p* < 0.0001).

Over time, the incidence rate in males varied from 50.44 cases per 100,000 person-years in 2020 to 82.06 cases per 100,000 person-years in 2017 with death rates varying from 22.82% in 2017 to 34.20% in 2016. The incidence rate in females varied from 45.56 per 100,000 person-years in 2020 to 74.16 per 100,000 person-years in 2017, with death rates varying from 25.40% in 2017 to 38.5% in 2016.

Males accounted for 52.41% of all stroke cases and 53% of all surviving cases, both proportions were significantly higher than females in both cases (*p* < 0.0001). However, males accounted for only 50% of all stroke cases resulting in deaths. Accordingly, females had a significantly higher death rate of 31.6% when compared to males with a death rate of 28.7% (*p* < 0.0001). Results are detailed in Table 3.

**Table 3:** Incidence and death rates for hospitalized cases of stroke by sex and year.

**Sex**	**Year**	**Hospitalized reported cases**	**Cases resulting in death**	**Total per year and sex**	**Case fatality rate**	**Incidence per 100,000 persons-year**	**95% Poisson confidence intervals per 100,000 persons-year**
**Male**	2015	4,611	1,587	6,198	25.61%	76.15	74.97–78.81
	2016	3,151	1,638	4,789	34.20%	57.95	56.86–60.19
	2017	5,313	1,571	6,884	22.82%	82.06	80.92–84.86
	2018	3,450	1,606	5,056	31.76%	59.4	58.35–61.68
	2019	3,618	1,648	5,266	31.30%	60.99	59.96–63.29
	2020	3,108	1,308	4,416	29.62%	50.44	49.47–52.48
Total males		23,251	9,358	32,609	28.7%	64.96	64.26–65.67
**Female**	2015	41,39	1,587	5,726	27.72%	70.35	67.89–71.52
	2016	2,617	1,639	4,256	38.51%	51.5	49.49–52.56
	2017	4,641	1,580	6,221	25.40%	74.16	71.63–75.29
	2018	3,065	1,577	4,642	33.97%	54.54	52.46–55.58
	2019	3,063	1,712	4,775	35.85%	55.3	53.21–56.33
	2020	2,730	1,259	3,989	31.56%	45.56	43.71–46.52
Total Females		20,255	9,354	29,609	31.6%	57.84	57.18–58.50
Total		43,506	18,712	62,218	30.07%	61.37	60.89–61.85

### Ethnic characteristics of stroke

Fifty two thousand and thirty three cases of stroke were distinguished by ethnic group in the hospital records. These groups corresponded to: Indigenous (16.78 per 100,000 persons-year *n* = 1,240/pop, 2.38% of all stroke cases), Afrodescendants (11.34 per 100,000 persons-year *n* = 857/pop; 1.64% of all stroke cases), White (9.49 per 100,000 persons-year *n* = 608; 1.15% of all stroke cases), Mestizo (30.9 per 100,000 persons-year *n* = 23,352; 93.56% of all stroke cases) and Montubio (8.34 per 100,000 persons-year, *n* = 648; 0.4% of all stroke cases). The number of cases and cumulative incidence varied significantly between ethnic groups (*p* < 0.0001).

Case fatality rates varied significantly among all ethnic groups (*p* < 0.0001). The highest fatality rate corresponded to the Montubio ethnic group (87.65%), followed by Afrodescendants (67.21%), White (62.35%), and then Indigenous (52.66%).The Mestizo group had the lowest case fatality rate.

### Ethnic and gender characteristics of stroke

Indigenous women represented 53.14% of all indigenous cases, the number of cases reported was significantly higher than the number of indigenous male cases (*p* = 0.02). Mestizo men represented 52.03% of all mestizo cases, the number of mestizo male cases was significantly higher than the corresponding proportional female case number (*p* < 0.0001). Montubio men represented 60.80% of Montubio stroke reported cases, the number of cases was also significantly higher than the proportional number of Montubio female cases (*p* < 0.0001). The other ethnic groups did not show significant differences between sexes for the total number of reported cases.

For reported cases that resulted in non-fatal cases, only the Mestizo ethnic group showed a significant difference between sexes (*p* < 0.0001) with males accounting for 53.15% of such cases. For cases that resulted in death, Indigenous and Montubios presented significant differences between sexes (*p* < 0.0001 for both groups). Indigenous females accounted for 58.03% and within the Montubio group, males accounted for 62.58% of cases resulting in death. Case fatality rates varied significantly between sexes for Indigenous, Afro-descendants, and Mestizo groups. Female indigenous people reported a case fatality rate of 57.51%; significantly higher (*p* = 0.04) when compared to 47.16% in males. In the Afro-descendant group, males had a significantly higher (*p* < 0.0001) case fatality rate with 70.86% when compared to females with a 63.55% case fatality rate. In the Mestizo group, females had a significantly higher case fatality rate (*p* < 0.0001) with 33.2% when compared to males with a case fatality rate of 30.05%.

### Age characterization of stroke by gender and ethnic group

The average age of stroke diagnosis was higher for females (66.45 y.o.) than for males (64.32 y.o.) in the general population but no significant statistical differences were found (*p* > 0.45). The average age of diagnosis did not differ significantly among ethnic groups (*p* > 0.9), nor by sex distinguished by ethnic groups. The only statistical difference that was observed was that between the average age of diagnosis of stroke in male Mestizo and female Mestizo populations (64.16 and 66.72, respectively, *p* < 0.0001).

For the average age of death from stroke, the average male age of death was significantly earlier (71.14 y.o.) than corresponding female figures (74.04 y.o.), not considering ethnic background *p* < 0.001). In terms of ethnic distinctions, Afrodescendents were prone to die significantly earlier with an average of 66.62 y.o. (*p* < 0.0001). Montubios had the highest average age of death with 76.1 y.o. Whites had the second highest average age of death with 74.6 y.o (*p* < 0.0001). Indigenous and Mestizos were not significantly different (*p* > 0.4) and were placed in the third and fourth place, respectively, when ranked by age of death. When considering sex and ethnic group together, male and female Afrodescendants had the lowest average age of death from stroke (66.78 and 66.44 respectively) (*p* < 0.0001). Female Montubio (77.86 y.o.), White females (75.84 y.o.), and male Montubio (75.06 y.o.) had the highest average ages of death from stroke (*p* < 0.05) when compared to other ethnic groups and sex. Details are shown in Table 4.

**Table 4:** Average ages for death from stroke and stroke diagnosis by sex and ethnic group.

	**Average age of death from stroke**			**Average age of stroke diagnosis**		
	**Males**	**Females**	**All**	**Males**	**Females**	**All**
**Indigenous**	71.14	74.04	72.83	64.78	66.21	65.46
**Afrodescendents**	66.78	66.44	66.62	64.13	64.88	64.54
**White**	73.19	75.89	74.57	63.28	66.93	65.07
**Mestizo**	69.41	73.36	71.39	64.16	66.72	65.36

### Burden of disease of stroke expressed in DALYs

The estimated burden of disease of stroke in Ecuador (2015–2020) varied from 1.468 to 2.991 DALY per 1,000 population on average depending on the scenario used for estimation. The Mestizo group contributed with the highest average proportion of DALYs per ethnic specific population followed by the Afrodescendant ethnic group. The Montubio ethnic group was the group with the lowest DALY average contribution, followed by the White ethnic group. The results are detailed in Table 5.

**Table 5:** 2015–2020 average DALYs per capita and relative burden by ethnicity.

**2015–2020 average**						
	**Total**	**Indigenous**	**Afrodescendent**	**White**	**Mestizo**	**Montubio**
Population in 1000s	16,897.765	1,187.897	1,215.177	1,029.468	12,153.767	1,246.130
**DALY/Pop/1000**						
No age weighting and no discount rate	2.991	1.372	1.464	0.759	3.288	0.868
Age weighting and no discount rate	2.090	0.973	1.060	0.484	2.308	0.5591
Age weighting and 3% discount rate	1.468	0.678	0.745	0.368	1.659	0.418
**Proportional to national average**						
No age weighting and no discount rate	1.000	0.459	0.489	0.254	1.099	0.290
Age weighting and no discount rate	1.000	0.466	0.507	0.232	1.104	0.267
Age weighting and 3% discount rate	1.000	0.462	0.508	0.251	1.130	0.285
**Contributions of YLD and YLL for DALYs** ^*^						
YLD/DALY	3%	2%	1%	2%	4%	0%
YLL/DALY	97%	98%	99%	98%	96%	100%

* YLD and YLL refer to years of life lost to disability and years of life lost to death, respectively.

YLLs represented 97% of all contributions to the burden of disease in DALYs for all ethnic groups. The ethnic groups with the highest YLL contribution were: Montubio (100%) and Afrodescendent (99%), while the Mestizo population had the lowest YLL contribution (96%).

## Discussion

This is the first study to conduct a sociodemographic characterization of stroke incidence and burden of disease in Ecuador using national hospital records. Stroke incidence for all groups for the years 2015–2020 was 61.37 per 100,000 person-years. This incidence is in line with that calculated through modeled data by the GBD 2019 Stroke Collaborators. Men had a higher incidence than women for that same period (64.96 vs. 57.84 per 100,000 person-years), while females had a significantly higher death rate (31.6%) than men (28.7%). Mestizos had the highest incidence (30.9 per 100,000 person-years), followed by Indigenous (16.78 per 100,000 persons-year) and Afro-descendants (11.34 per 100,000 persons-year). It is likely, however, that these figures are skewed by poor access to care and therefore diagnosis in minority groups. Montubios and Afrodescendants, both recognized as minority ethnic groups, had the highest case fatality rates (i.e., 87.65 and 67.21%, respectively). When looking at the intersection of gender and ethnicity/race, Indigenous and Montubio females appeared to be at risk, accounting for 62.58 and 58.03% of cases resulting in death respectively. In addition, Afrodescendants were prone to die significantly earlier with an average of 66.62 y.o.

The estimated stroke burden of disease in hospital admitted Ecuadorians (2015–2020) ranged from 146.8 to 299.1 DALY per 100,000 population on average. The highest average proportion of DALYs per ethnic group was for Mestizos followed by Afro-descendants. These calculations fall below those of the GBD 2019 Stroke Collaborators' estimates for Ecuador for all stroke types., However, even our conservative estimate reveals a large loss of life quality due to stroke in Ecuador. Again, it is possible that differential access to care had an impact on these results. These relative results bear some similarities with research in other regions regarding sociodemographic differences in stroke outcomes (37), however the absolute magnitude of the years of lives lost as a proportion of the population is estimated to be much smaller than other studies. This is expected to be due to the use of hospital records as source data, which only captures hospitalized cases and not the cases that do not make it to hospital. The likely underestimation of DALYs in this study suggests that there exists a large number of stroke cases that do not make it to the hospital system at all.

Ethnic/racial disparities have been documented in prior research, with Afrodescendants showing poorer outcomes in multiple regions. Various causes have been suggested for this phenomenon, including lack of access to adequate care, inadequate control of medical risk factors, poor medication adherence, various psychosocial determinants of health, and genetic predisposition among others (38, 39). A review of stroke risk factors for Whites and African-Americans in the U.S. found that the latter often receive inadequate pharmacological therapy for hyperlipidemia and hypertension (40), which is particularly problematic in light of that group's higher incidence of hypertension when compared to other racial/ethnic groups (41). Indeed, African-Americans have also been found to have a higher risk for recurrent stroke than Whites, which may be mediated by poor control of risk factors (10).

Results from a survey in Cuba, where ethnic differences in socioeconomic factors and in access and quality of care are less marked, (42), found no differences in prevalence of hypertension between black and white males (43). Interestingly, white females showed a slightly lower prevalence than black females, which corresponded with somewhat better treatment and control rates for the former group, thereby underscoring the substantive role that proper medical care plays in neutralizing stroke risk factors. Consistent with these findings, cardiovascular risk factors for populations of African origins vary across regions, with higher Framingham risk scores associated with higher socioeconomic development (44), further suggesting environmental factors play a significant role in outcome disparities. However, research also exists that supports a genetic contribution to stroke incidence. Traylor et al. (45) found a substantial genetic contribution for stroke risk in populations of African descent. Consistent with this finding, Carty et al. (39) found a novel genetic variant associated with stroke in African-Americans in a meta-analysis consisting of 14,746 cases of stroke in that population.

Research into stroke and indigenous people of the Americas is limited. A somewhat recent systematic review (46) looking at stroke in native Americans and Alaskan natives in the United States showed a higher prevalence of stroke and cerebrovascular risk factors than in other US populations. Furthermore, the studied groups had strokes at a younger age than non-Hispanic Whites. The authors don't provide a rationale for the findings other than noting historical changes to diet in this population and a history of trauma brought about by colonial practices. To the best of the author's knowledge, studies looking at stroke characteristics in indigenous populations of Latin America are not currently available.

There is ample research regarding stroke and gender showing men tend to have a higher incidence and prevalence than women (18). This is consistent with results from the current study, although female mortality was higher and the literature regarding this is mixed, with some studies showing higher mortality in women and others finding no differences (15, 16, 18). Most of these studies took place in developed regions; however, a recent review of sex differences in stroke in a Latin American registry (47), found worse functional outcomes and higher mortality rates for women, consistent with the current study.

Reasons for the current results regarding ethnic disparities are understandably unclear given the paucity of data. In light of research from other regions, nevertheless, it is likely that environmental factors play a large role, while genetic contributions should not be discarded. Given that the contribution of stroke risk factors to the burden of disease (behavioral, environmental, and occupational) has already been calculated by GBD in 2019, it is of interest to assess the differences in stroke risk factors between ethnic/racial groups, in order to determine how they might contribute to the variations observed in this study (33). In the study performed by White et al. (48), which included a total of 3,020 participants from 8 countries, encompassing Ecuador, Peru, Chile and Argentina, significant differences were reported by race/ethnicity in lifestyle behaviors including smoking status, alcohol use, and regular exercise. Moreover, the results showed that Hispanics and Afrodescendants were more likely to have a history of hypertension and diabetes.

Accompanying that, one must consider whether access and quality of medical care varies per ethnic/racial groups. In a study by the PAHO in 2008 (49), it was demonstrated that the ethnic group with the least access to healthcare is the indigenous population (12% for males and 14% for females), followed by the Afro-Ecuadorian population (15% for males and 19% for females). The numbers reflect that the main factor that affects health status is the level of poverty, which is correlated with ethnic identity in Ecuador. In the aforementioned publication, it was also shown that the population with the highest poverty index is the indigenous population, with 68% living below the poverty line and from these, 40% are living in extreme poverty. The Afro-Ecuadorian population is placed second, with 43% living under the poverty line and from this, 11% are living in extreme poverty (49). The link between poverty, limited access to healthcare and stroke prevalence has been demonstrated in Brazil (50) and Bolivia (51) as well. In addition, the published studies by the GBD Stroke Collaborators in 2019 evidenced that the burden of stroke decreases in regions with higher sociodemographic indexes (33). Furthermore, in Ecuador, around 36% of the population (over 6 million people) live in rural regions of the country (52). Of those that live in rural areas, 43% live in poverty compared to the 15.9% in urban areas. In Ecuador, 86% of public health care providers and 96% of private practice providers operate in urban areas. Consequently, high-quality, preventive stroke care, treatment and rehabilitation are not readily available for subsets of the population with lower income. A study performed in Southern Ecuador in 2022 identified that the main health care access barriers in rural communities are the cost of transportation and medication, lack of available appointments with healthcare providers, distance to clinics and hospitals, low level of health literacy, preference of use of traditional medications and self-treatment, and lack of preventative measures (53).

Another risk factor that needs to be considered is the spatial distribution of Tripanosoma cruzi that expands across Latin America. *T. cruzi* is the parasite responsable of Chagas Disease (CD), ranked as one of the most serious infectious diseases in the region due to the important health, economic, and social burdens it entails (54). It is mainly associated with poverty and rural areas, due to precarious housing conditions and therefore, considered a neglected tropical disease. Approximately, 30% of infected individuals develop chagasic cardiomyopathy starting with conduction system blocks and ventricular abnormalities that lead to life threatening arrythmia, bradycardia, dilated cardiomyopathy, congestive heart failure, and emboli formation (55). Stroke had been an unrecognized complication of Chagas disease until recently (56). This may also account for the differences in stroke prevalence between race/ethnicity found in this study.

Of particular concern within all findings are certain results for the Afrodescendant group. They have a very elevated case fatality rate, die earliest from stroke, and have the second highest burden of disease, in spite of lower representation in hospital discharge records. Taken together, these statistics suggest they are a particularly vulnerable group, which might require public health policies to address their needs specifically. Given results from other regions indicating poor blood pressure control and high cholesterol levels in Afrodescendants, these variables should be assessed routinely in this population and interventions developed to improve these health indicators. Lastly, higher death rates in females, which are substantially higher when intersected with certain minority ethnic/racial groups, also raise concern, and require further investigation. Prior research in other regions suggests stroke symptoms may vary by gender and awareness of modifiable risk factors in women is poor. Assuming the same may hold for Ecuador, increased education might improve outcomes for women.

## Limitations

Limitations of this study relate to the data source and its ability to be representative. Characteristics of the data set include patient's self-identification of ethnicity/race, thus relying on a patient's understanding of their racial background which may or may not be correct. This is particularly problematic in Latin American societies where colorism, the practice of discrimination based on skin color, may render identification with indigenous and/or Afrodescendent groups socially undesirable. Another limitation is that sample sizes for some of the ethnic/racial groups were small, and consequently comparisons may have been underpowered. However, differences in stroke-diagnosis likelihood were appreciated despite this limitation. Finally, within the hospital records, the cause of death was undetermined in greater numbers for certain ethnic/racial groups, possibly leading to a sub estimation of stroke related deaths and thus burden of disease.

The literature shows that the quality of hospital discharge records varies across systems, with some studies being deemed to exhibit adequate accuracy (57, 58) and others to display questionable integrity (59). Accordingly, the calculation of stroke incidence in this study should be taken with caution and probably does not represent the real incidence in the population of Ecuador at large. It is possible that coding inaccuracies are also present in the records used in the analysis. However, it is important to note that the objective of the study is not to document the incidence of stroke in the general population, but within that recorded in hospital discharge records. While there is no direct evidence, it is also possible that the quality of hospital discharge records and the consistency with which certain patient characteristics are recorded may differ by region. Where ethnic composition of the population also varies by region, there may be some underestimation of hospital treated stroke incidence by ethnicity. As such, this analysis is vulnerable to differences in access to care and/or treatment seeking behavior between ethnic/racial groups. It is possible that certain ethnic/racial groups are less likely to seek or receive care for emergent conditions such as stroke, decreasing their representation in hospital records. Finally, some discharge events may represent instances of the same patient being hospitalized at different points in time and it is therefore possible that one racial group has a higher likelihood of being discharged with a stroke diagnosis due to a subset of that population being hospitalized repeatedly for that condition. Nevertheless, it remains true that there is a disparity between ethnic/racial and gender groups per the current results even in this scenario. Furthermore, in spite of their limitations, clinical registries are commonly used in epidemiological studies and considered useful in improving quality of medical care and the overall health of the population (60–65).

Given the lack of data regarding ethnic/racial disparities in stroke outcome, further research in this area is recommended, particularly a community based prospective approach to overcome the limitations of this study and confirm the ethnic/racial disparities found here. The aforementioned Atahualpa project from Del Brutto et al. (66) stands out as a notable example of the type of research required, that is prospective, community based, and includes medical imaging for corroboration of survey results, although sampling would have to extend to different regions targeting the ethnic/racial diversity of the country. As the author's note, the region sampled was specifically selected due to the population's homogeneity, including regarding ethnicity/race. Lastly, studies examining differences in stroke risk factors between ethnic/racial groups, such as hypertension and hyperlipidemia, are also warranted as they may lead to effective intervention strategies. Finally, research targeted at identifying reasons for sex-based stroke disparities is sorely needed, which should act as a basis for improved outcomes for women.

## Data availability statement

The original contributions presented in the study are included in the article/Supplementary material, further inquiries can be directed to the corresponding authors.

## Author contributions

All authors listed have made a substantial, direct, and intellectual contribution to the work and approved it for publication.

## Footnotes

^1^Institute for Health Metrics and Evaluation (IHME) (32); Global Burden of Disease Study 2019 (33) Disability Weights, accessed 21st of March, 2022: https://ghdx.healthdata.org/record/ihme-data/gbd-2019-disability-weights.

^2^See Appendix 1 for a table with each stroke type and the disability weight estimated to correspond.
