# Supplementary material for: Ethnodemographic characterization of stroke incidence and burden of disease in hospital discharge records in Ecuador
Source: Front Neurol. 2023 Feb 8;14:1059169. doi: 10.3389/fneur.2023.1059169 (PMC9945224; doi:10.3389/fneur.2023.1059169)
Supplement: Supplementary file 1 [file Table_1.docx]

**Annexes**

**Annex. 1**

| Ethnic group | Sex | Year | Live cases | Cases resulting in death | Total cases | Case fatality rate rate | Incidence rate per 100,000 person-years | 95% Poisson Confidence intervals |
| --- | --- | --- | --- | --- | --- | --- | --- | --- |
| Indigenous | Males | 2015 | 42 | 39 | 81 | 48.15% | 14.16 | 11.35-17.76 |
|  |  | 2016 | 37 | 35 | 72 | 48.61% | 12.39 | 9.79-15.76 |
|  |  | 2017 | 73 | 49 | 122 | 40.16% | 20.69 | 17.35-24.95 |
|  |  | 2018 | 48 | 53 | 101 | 52.48% | 16.88 | 13.89-20.72 |
|  |  | 2019 | 62 | 48 | 110 | 43.64% | 18.12 | 15.04-22.07 |
|  |  | 2020 | 45 | 50 | 95 | 52.63% | 15.43 | 12.62-19.06 |
|  | Females | 2015 | 37 | 50 | 87 | 57.47% | 15.20 | 12.06-18.58 |
|  |  | 2016 | 28 | 68 | 96 | 70.83% | 16.52 | 13.26-19.99 |
|  |  | 2017 | 81 | 69 | 150 | 46.00% | 25.44 | 21.32-29.56 |
|  |  | 2018 | 34 | 55 | 89 | 61.80% | 14.87 | 11.83-18.12 |
|  |  | 2019 | 58 | 66 | 124 | 53.23% | 20.43 | 16.82-24.11 |
|  |  | 2020 | 42 | 71 | 113 | 62.83% | 18.36 | 14.98-21.85 |
| Afroecuatoriano | Males | 2015 | 15 | 70 | 85 | 82.35% | 14.52 | 11.71-18.13 |
|  |  | 2016 | 12 | 31 | 43 | 72.09% | 7.24 | 5.29-9.84 |
|  |  | 2017 | 27 | 70 | 97 | 72.16% | 16.08 | 13.17-19.81 |
|  |  | 2018 | 24 | 19 | 43 | 44.19% | 7.02 | 5.14-9.56 |
|  |  | 2019 | 30 | 89 | 119 | 74.79% | 19.17 | 16.04-23.17 |
|  |  | 2020 | 17 | 25 | 42 | 59.52% | 6.67 | 4.86-9.11 |
|  | Females | 2015 | 21 | 55 | 76 | 72.37% | 13.0 | 10.14-16.10 |
|  |  | 2016 | 16 | 44 | 60 | 73.33% | 10.1 | 7.63-12.87 |
|  |  | 2017 | 45 | 51 | 96 | 53.13% | 15.9 | 12.77-19.25 |
|  |  | 2018 | 30 | 29 | 59 | 49.15% | 9.6 | 7.27-12.31 |
|  |  | 2019 | 24 | 66 | 90 | 73.33% | 14.5 | 11.54-17.64 |
|  |  | 2020 | 20 | 27 | 47 | 57.45% | 7.5 | 5.43-9.83 |
| White | Males | 2015 | 2 | 40 | 42 | 95.24% | 8.47 | 6.16-11.56 |
|  |  | 2016 | 18 | 45 | 63 | 71.43% | 12.51 | 9.71-16.17 |
|  |  | 2017 | 38 | 36 | 74 | 48.65% | 14.48 | 11.49-18.36 |
|  |  | 2018 | 21 | 21 | 42 | 50.00% | 8.10 | 5.90-11.06 |
|  |  | 2019 | 18 | 33 | 51 | 64.71% | 9.70 | 7.30-12.88 |
|  |  | 2020 | 19 | 9 | 28 | 32.14% | 5.25 | 3.53-7.67 |
|  | Females | 2015 | 18 | 47 | 65 | 72.31% | 13.11 | 10.03-16.56 |
|  |  | 2016 | 15 | 42 | 57 | 73.68% | 11.32 | 8.50-14.53 |
|  |  | 2017 | 29 | 36 | 65 | 55.38% | 12.72 | 9.72-16.06 |
|  |  | 2018 | 21 | 21 | 42 | 50.00% | 8.10 | 5.78-10.84 |
|  |  | 2019 | 9 | 36 | 45 | 80.00% | 8.55 | 6.18-11.34 |
|  |  | 2020 | 19 | 10 | 29 | 34.48% | 5.44 | 3.61-7.73 |
| Mestizo | Males | 2015 | 3496 | 1275 | 4771 | 26.72% | 81.50 | 79.95-84.63 |
|  |  | 2016 | 2395 | 1371 | 3766 | 36.40% | 63.36 | 61.94-66.04 |
|  |  | 2017 | 4191 | 1222 | 5413 | 22.58% | 89.72 | 88.20-93.04 |
|  |  | 2018 | 2695 | 1359 | 4054 | 33.52% | 66.22 | 64.84-68.97 |
|  |  | 2019 | 2521 | 1267 | 3788 | 33.45% | 61.00 | 59.67-63.61 |
|  |  | 2020 | 2422 | 1119 | 3541 | 31.60% | 56.23 | 54.95-58.71 |
|  | Females | 2015 | 3189 | 1308 | 4497 | 29.09% | 76.82 | 73.88-78.35 |
|  |  | 2016 | 2030 | 1357 | 3387 | 40.06% | 56.98 | 54.55-58.37 |
|  |  | 2017 | 3627 | 1266 | 4893 | 25.87% | 81.10 | 78.07-82.59 |
|  |  | 2018 | 2410 | 1375 | 3785 | 36.33% | 61.83 | 59.28-63.20 |
|  |  | 2019 | 2163 | 1366 | 3529 | 38.71% | 56.83 | 54.42-58.14 |
|  |  | 2020 | 2198 | 1063 | 3261 | 32.60% | 51.78 | 49.51-53.05 |
| Montubio | Males | 2015 | 8 | 49 | 57 | 85.96% | 9.47 | 7.25-12.39 |
|  |  | 2016 | 1 | 61 | 62 | 98.39% | 10.15 | 7.86-13.14 |
|  |  | 2017 | 9 | 64 | 73 | 87.67% | 11.77 | 9.32-14.95 |
|  |  | 2018 | 10 | 70 | 80 | 87.50% | 12.71 | 10.19-15.99 |
|  |  | 2019 | 4 | 65 | 69 | 94.20% | 10.81 | 8.50-13.83 |
|  |  | 2020 | 9 | 44 | 53 | 83.02% | 8.19 | 6.20-10.83 |
|  | Females | 2015 | 1 | 40 | 41 | 97.56% | 6.81 | 4.85-9.16 |
|  |  | 2016 | 4 | 38 | 42 | 90.48% | 6.87 | 4.91-9.21 |
|  |  | 2017 | 21 | 44 | 65 | 67.69% | 10.48 | 8.01-13.24 |
|  |  | 2018 | 6 | 34 | 40 | 85.00% | 6.36 | 4.50-8.57 |
|  |  | 2019 | 5 | 31 | 36 | 86.11% | 5.64 | 3.91-7.73 |
|  |  | 2020 | 6 | 24 | 30 | 80.00% | 4.63 | 3.10-6.55 |
| Total |  |  | 34516 | 17517 | 52033 |  |  |  |

**Annex 2**

| **2015** | | | | | | |
| --- | --- | --- | --- | --- | --- | --- |
|  | **Total** | **Indigenous** | **Afrodescendent** | **White** | **Mestizo** | **Montubio** |
| Population in 1000s | 16278.844 | 1144.387 | 1170.669 | 991.761 | 11708.606 | 1203.453 |
| **DALY/Pop/1000** | | | | | | |
| No age weighting and no discount rate | 3.186 | 1.302 | 2.013 | 1.323 | 3.474 | 0.845 |
| Age weighting and no discount rate | 2.278 | 0.986 | 1.539 | 0.930 | 2.517 | 0.518 |
| Age weighting and 3% discount rate | 1.576 | 0.668 | 1.035 | 0.663 | 1.770 | 0.401 |
| **PROPORTIONAL TO NATIONAL AVERAGE** | | | | | | |
| No age weighting and no discount rate | 1.000 | 0.409 | 0.632 | 0.415 | 1.091 | 0.265 |
| Age weighting and no discount rate | 1.000 | 0.433 | 0.676 | 0.408 | 1.105 | 0.227 |
| Age weighting and 3% discount rate | 1.000 | 0.424 | 0.657 | 0.421 | 1.123 | 0.255 |
| **2016** | | | | | | |
|  | **Total** | **Indigenous** | **Afrodescendent** | **White** | **Mestizo** | **Montubio** |
| Population in 1000s | 16528.730 | 1161.954 | 1188.639 | 1006.985 | 11888.337 | 1221.927 |
| **DALY/Pop/1000** | | | | | | |
| No age weighting and no discount rate | 3.182 | 1.183 | 1.251 | 1.129 | 3.470 | 0.927 |
| Age weighting and no discount rate | 2.269 | 0.781 | 0.885 | 0.748 | 2.391 | 0.602 |
| Age weighting and 3% discount rate | 1.564 | 0.576 | 0.623 | 0.551 | 1.737 | 0.442 |
| **PROPORTIONAL TO NATIONAL AVERAGE** | | | | | | |
| No age weighting and no discount rate | 1.000 | 0.372 | 0.393 | 0.355 | 1.091 | 0.291 |
| Age weighting and no discount rate | 1.000 | 0.344 | 0.390 | 0.330 | 1.054 | 0.265 |
| Age weighting and 3% discount rate | 1.000 | 0.368 | 0.399 | 0.352 | 1.111 | 0.283 |
| **2017** | | | | | | |
|  | **Total** | **Indigenous** | **Afrodescendent** | **White** | **Mestizo** | **Montubio** |
| Population in 1000s | 16776.977 | 1179.405 | 1206.491 | 1022.109 | 12066.890 | 1240.279 |
| **DALY/Pop/1000** | | | | | | |
| No age weighting and no discount rate | 3.018 | 1.470 | 1.726 | 0.783 | 3.316 | 1.143 |
| Age weighting and no discount rate | 2.090 | 1.049 | 1.222 | 0.437 | 2.347 | 0.768 |
| Age weighting and 3% discount rate | 1.485 | 0.707 | 0.879 | 0.366 | 1.688 | 0.559 |
| **PROPORTIONAL TO NATIONAL AVERAGE** | | | | | | |
| No age weighting and no discount rate | 1.000 | 0.487 | 0.572 | 0.259 | 1.099 | 0.379 |
| Age weighting and no discount rate | 1.000 | 0.502 | 0.585 | 0.209 | 1.123 | 0.367 |
| Age weighting and 3% discount rate | 1.000 | 0.476 | 0.592 | 0.246 | 1.137 | 0.376 |
| **2018** | | | | | | |
|  | **Total** | **Indigenous** | **Afrodescendent** | **White** | **Mestizo** | **Montubio** |
| Population in 1000s | 17023.408 | 1196.729 | 1224.213 | 1037.123 | 12244.136 | 1258.497 |
| **DALY/Pop/1000** | | | | | | |
| No age weighting and no discount rate | 3.062 | 1.449 | 0.814 | 0.408 | 3.515 | 0.828 |
| Age weighting and no discount rate | 2.117 | 1.073 | 0.618 | 0.222 | 2.435 | 0.484 |
| Age weighting and 3% discount rate | 1.496 | 0.729 | 0.421 | 0.188 | 1.771 | 0.387 |
| **PROPORTIONAL TO NATIONAL AVERAGE** | | | | | | |
| No age weighting and no discount rate | 1.000 | 0.473 | 0.266 | 0.133 | 1.148 | 0.270 |
| Age weighting and no discount rate | 1.000 | 0.507 | 0.292 | 0.105 | 1.150 | 0.229 |
| Age weighting and 3% discount rate | 1.000 | 0.487 | 0.282 | 0.126 | 1.184 | 0.259 |
| **2019** | | | | | | |
|  | **Total** | **Indigenous** | **Afrodescendent** | **White** | **Mestizo** | **Montubio** |
| Population in 1000s | 17267.986 | 1213.923 | 1241.801 | 1052.023 | 12420.049 | 1276.578 |
| **DALY/Pop/1000** | | | | | | |
| No age weighting and no discount rate | 3.131 | 1.564 | 2.128 | 0.694 | 3.226 | 0.906 |
| Age weighting and no discount rate | 2.151 | 1.117 | 1.458 | 0.413 | 2.236 | 0.616 |
| Age weighting and 3% discount rate | 1.525 | 0.775 | 1.067 | 0.328 | 1.609 | 0.447 |
| **PROPORTIONAL TO NATIONAL AVERAGE** | | | | | | |
| No age weighting and no discount rate | 1.000 | 0.499 | 0.680 | 0.222 | 1.030 | 0.289 |
| Age weighting and no discount rate | 1.000 | 0.519 | 0.678 | 0.192 | 1.040 | 0.287 |
| Age weighting and 3% discount rate | 1.000 | 0.508 | 0.700 | 0.215 | 1.055 | 0.293 |
| **2020** | | | | | | |
|  | **Total** | **Indigenous** | **Afrodescendent** | **White** | **Mestizo** | **Montubio** |
| Population in 1000s | 17510.643 | 1230.981 | 1259.251 | 1066.807 | 12594.581 | 1294.517 |
| **DALY/Pop/1000** | | | | | | |
| No age weighting and no discount rate | 2.396 | 1.260 | 0.879 | 0.270 | 2.756 | 0.560 |
| Age weighting and no discount rate | 1.660 | 0.832 | 0.660 | 0.190 | 1.944 | 0.364 |
| Age weighting and 3% discount rate | 1.175 | 0.608 | 0.458 | 0.136 | 1.394 | 0.272 |
| **PROPORTIONAL TO NATIONAL AVERAGE** | | | | | | |
| No age weighting and no discount rate | 1.000 | 0.526 | 0.367 | 0.113 | 1.150 | 0.234 |
| Age weighting and no discount rate | 1.000 | 0.501 | 0.398 | 0.115 | 1.171 | 0.219 |
| Age weighting and 3% discount rate | 1.000 | 0.518 | 0.390 | 0.116 | 1.187 | 0.232 |
